# Dry January and beyond

**DOI:** 10.1016/j.eclinm.2025.103082

**Published:** 2025-01-21

**Authors:** eClinicalMedicine

With the start of a new year also comes some reflection on the past year and maybe even some desire for change. Health-related new year's resolutions usually stretch from more exercise to a healthier diet and in some cases less alcohol consumption. Such resolutions can be achieved mostly by modifying our daily habits and lifestyle, which requires discipline and endurance. In an effort to help and encourage people to change their drinking habits, Dry January was founded by Alcohol Change UK, a charity that focuses on reducing alcohol-related harm in the UK. The campaign encourages people to abstain from drinking alcohol for the entire month of January. It was launched in 2013, originating by Emily Robinson in 2011 when she signed up for her first half marathon and gave up drinking in January. In the following year, she joined Alcohol Change UK and her inspiration led to the first official Dry January campaign in 2013. Since its inception, Dry January has grown in popularity from 4000 official participants in 2013 to 175000 in 2023. The campaign has inspired similar initiatives in other countries. In the USA, many bars, restaurants, and beverage companies have started to support Dry January by offering a variety of non-alcoholic drinks and mocktails to accommodate those participating in the initiative.

The negative effect of alcohol consumption on long-term human health has been known for a long time. Excessive alcohol intake is associated with an increased risk in developing liver diseases, cancer, obesity, and cardiovascular diseases, but it also increases the risk for harmful injuries from falls, violence, and motor vehicle crashes. Alcohol can damage the heart, which can lead to cardiomyopathy, arrythmia, stroke, and high blood pressure. There is a strong scientific link between alcohol and several types of cancers with the strongest association for head and neck, esophageal, liver, breast, and colorectal cancers. Underage drinking as well as excessive drinking during childhood, adolescence, and early adulthood is associated with many immediate and longer-term health problems. Drinking at a young age can cause long-term harm and damage to the brain and can lead to alcohol use disorders later in life. It also strongly affects decision-making which can lead to injuries or death for themselves or others. In fact, injuries account for the majority of disability-adjusted life years lost in individuals aged 15–39 years. Overall in 2019, alcohol consumption accounted for 2.07 million deaths of males and 374,000 deaths of females, globally.

Beyond campaigns like Dry January, global organisations such as the WHO are also actively promoting alcohol-free social spaces, including alcohol-free parties and bars, as part of their strategy to reduce alcohol-related harm. To further combat the negative effect of alcohol the WHO member states endorsed the global alcohol action plan aiming to reduce the harmful use of alcohol and its associated health and social issues worldwide. The action plan was released in June 2024 in support of the achievement of Sustainable Development Goals by promoting healthier communities. It outlines a comprehensive approach to tackle the negative impacts of alcohol consumption through a combination of policy measures, public health strategies, and community engagement. Key components of the plan include the implementation of effective alcohol policies with a focus on reducing alcohol-related harm. Furthermore, to advocate for the adoption of strategies the action plan includes taxation, regulation of availability, and restrictions on marketing and to further increase public awareness about the risk.

On January 3, 2025, the US Surgeon General Vivek Murthy issued a new advisory on the link between alcohol and cancer risk. The advisory emphasises that even moderate drinking can elevate the risk of various cancers. Furthermore, the advisory aims to inform the public and health-care providers about the potential dangers of alcohol, promoting healthier lifestyle choices to reduce cancer risk. Specifically, the advisory makes recommendations for health warning label on alcohol-containing beverages to now include cancer risk.

New research now underscores the benefits of alcohol abstinence on the reduction of cancer risk. Alcohol rehabilitation, which is linked to at least temporary reduction or cessation of alcohol consumption, even for a short term of 8–25 days led to 40% relative reductions in the long-term risk for alcohol-associated cancer. This provides scientific support for the efforts of Dry January and highlights that even no alcohol for 1 month can positively affect long-term health. Meanwhile, ongoing large, non-inferiority randomised controlled trials can provide even further solid evidence on the health benefits of alcohol abstinence compared with moderate drinking.

Overall, Dry January is a global project raising the awareness of alcohol-related health risks and provides an outlet for an active debate on conscious decision making and responsible drinking habits. We can say that less is always better, but we should not fall into false security by being good for 1 month out of the whole year and should rather aim for a balanced and responsible approach for the whole year.

